# Enrichment of Gluten‐Free Fresh Pasta With Cricket Flour: Technological and Nutritional Quality

**DOI:** 10.1002/fsn3.72213

**Published:** 2026-08-04

**Authors:** Carlos Gabriel Arp, Stefano Zardetto, Alyssa Hidalgo, Gabriella Pasini

**Affiliations:** ^1^ Department of Agronomy, Food, Natural Resources, Animals and Environment University of Padova Legnaro Italy; ^2^ Centro de Investigación y Desarrollo en Ciencia y Tecnología de Alimentos (CIDCA) Facultad de Ciencias Exactas‐Universidad Nacional de La Plata, Comisión de Investigaciones Científicas de la Provincia de Buenos Aires, Consejo Nacional de Investigaciones Científicas y Técnicas La Plata Argentina; ^3^ Gruppo Voltan, Assicurazione Qualità & R&D Olmo di Martellago (VE) Italy; ^4^ Department of Food, Environmental and Nutritional Sciences (DeFENS) Università Degli Studi di Milano Milan Italy

**Keywords:** *Acheta domesticus*, amino acids, composition, gluten‐free pasta, in vitro digestibility

## Abstract

The enrichment of gluten‐free fresh pasta with commercial cricket flour ( *Acheta domesticus* ) was investigated as a strategy to improve nutritional and technological quality. Pasta formulations containing 0%, 5.8%, and 10% cricket flour were studied. Cricket flour significantly increased protein and mineral content while reducing lipids and carbohydrates. Protein determined by amino acid count, rather than total nitrogen analysis, avoided overestimation due to chitin. Cooking trials revealed shorter optimal cooking times and lower water absorption in enriched samples, while cooking loss remained within acceptable limits. Textural analysis showed reduced firmness in uncooked pasta, although cooking minimized differences among formulations. Cricket flour imparted a darker hue to uncooked pasta that diminished after cooking, consistent with pigment leaching and degradation. Starch digestibility profiles showed moderate effects depending on enrichment level. Protein digestibility remained high (> 80%), and amino acid profiling demonstrated improved lysine content. Consumers expressed doubt or negativity toward uncooked samples with 10% cricket flour but responded favorably to 0% and 5.8% formulations. After cooking, all samples received mainly positive evaluations. Overall, *A. domesticus* flour proved to be a promising ingredient for enhancing protein quality and sustainability of gluten‐free fresh pasta while maintaining acceptable technological performance.

## Graphical abstract

Gluten‐free fresh pasta was formulated with increasing levels of *Acheta domesticus* flour (0%–10%). Nutritional composition, cooking performance, color, texture, and in vitro digestibility were evaluated. Consumer perception revealed acceptable sensory quality up to 5.8% inclusion, highlighting cricket flour as a promising ingredient for protein‐enriched gluten‐free pasta.

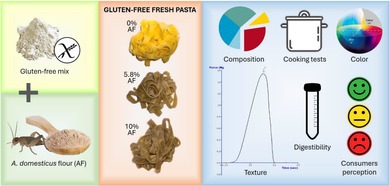

## 1 Introduction

Celiac disease is a chronic autoimmune enteropathy triggered by gluten ingestion in genetically predisposed individuals, with a global prevalence of 1%–2% and higher rates in specific populations (Lebwohl et al. 2015; Manninen et al. 2025). While adherence to a gluten‐free diet remains the only effective therapy (Green and Cellier 2007), the technological and nutritional limitations of gluten‐free fresh pasta formulations are particularly critical: they often rely on starches and gums, resulting in products with low protein density and poor micronutrient profiles (Keyes et al. 2026; Mostafa et al. 2025). This imbalance increases risks of secondary conditions such as type 2 diabetes, cardiovascular disease, and osteoporosis (Green and Cellier 2007).

Recent research has explored sustainable alternatives to improve gluten‐free products, including shrimp shell powder, seaweed, and banana waste flours, which enhance nutritional quality and reduce environmental impact (Hedwig et al. 2026; Philip and Rajasree 2026; Yerlikaya et al. 2025). These studies highlight the growing interest in valorizing novel raw materials for gluten‐free formulations.

Among sustainable protein sources, *Acheta domesticus* (house cricket) has gained attention due to its balanced amino acid profile, digestibility, and favorable lipid composition (van Huis et al. 2013; Zielińska et al. 2026). *A. domesticus* is widely farmed, commercially available, and already approved for human consumption in several jurisdictions, making it a practical candidate for industrial applications. Its selection in this study is motivated by both nutritional potential and regulatory feasibility.

Edible insects more broadly have been recognized as nutrient‐rich and environmentally sustainable, with reviews by van Huis et al. (2013) and Zielińska et al. (2026) emphasizing their role in diversifying diets and reducing ecological footprints. In pasta systems, insect powders have been shown to improve protein content, mineral availability, and sensory properties (Arp and Pasini 2024).

Previous studies have tested insect incorporation in diverse gluten‐free matrices: *Bombyx mori* in buckwheat pasta (Biró et al. 2019), *Gryllus bimaculatus* in rice and tapioca noodles (Wannasupchue and Wongthahan 2024), and *A. domesticus* in riceberry rice noodles (Musika et al. 2024). However, these systems differ in starch composition, protein interactions, and consumer expectations compared to fresh pasta, limiting direct generalization. The unresolved gap lies in understanding how cricket powder performs in industrial gluten‐free fresh pasta formulations, where both texture and nutritional enrichment must be balanced.

This study specifically evaluates the incorporation of commercial, partially defatted *A. domesticus* powder into gluten‐free fresh pasta. The rationale is to reduce carbohydrates by replacing corn starch and partially substituting corn flour, thereby addressing glycemic concerns, while also reducing oil content to achieve lower caloric density and improved lipid quality, taking advantage of the lipid content of cricket powder.

The novelty of this work lies in testing gluten‐free fresh pasta formulations with cricket powder in an industrial system, and in simultaneously replacing both starch and oil, an approach not previously reported. This strategy aims to obtain a healthier product for the celiac population by improving protein and lipid profiles while reducing glycemic load. The study addresses both the technological and nutritional aspects of these formulations, complemented by an evaluation of consumer sensory perception to ensure practical relevance.

## 2 Materials and Methods

### 2.1 Materials

The control gluten‐free fresh pasta sample was prepared using a gluten‐free mix consisting of pre‐gelatinized corn flour (according to the supplier's technical datasheet, as is: 13.5% moisture, 7% protein, 1% lipids, 76% carbohydrates, 2.5% fiber) (Molino Favero S.r.l., Italy), corn starch (according to the supplier's technical datasheet, as is: 14% moisture, 0.3% protein, 87.7% carbohydrates) (Macor di Trucazzano S.r.l., Itlay), a functional blend for gluten‐free pasta (corn and rice starch, pea protein, pea fiber, and xanthan and guar gum) (Qores S.r.l., Italy), sunflower oil (Pellegrini S.r.l., Italy), and tap water (hardness: 40°f; pH: 7.1; total dissolved solids: 280 mg/L). Enriched gluten‐free pasta samples were prepared using partially defatted cricket powder from *A. domesticus* (according to the supplier's technical datasheet, as is: 4.5% moisture, 74% protein, 12% lipids, 9.16% fiber) (Italian Cricket Farm S.r.l., Italy). *A. domesticus* powder was used in two levels (5.8% and 10%) to completely replace corn starch, partially reduce corn flour, and decrease sunflower oil in the gluten‐free mix, relative to the control. This formulation strategy was intended to increase protein content, lower carbohydrates, and theoretically reduce lipid levels by ~34% respect to the control. The control formulation, as well as the two cricket powder‐enriched formulations tested in this study, are presented in Table 1.

**TABLE 1:** Gluten‐free pasta formulations.

Ingredients	Control	AD5.8	AD10
Gluten‐free mix	60.1	50.8	46.4
Corn flour	38.0	35.1	30.6
Corn starch	6.2	0.0	0.0
Functional blend	15.8	15.8	15.8
*A. domesticus* flour	0.0	5.8	10.0
Sunflower oil	3.9	1.8	1.3
Water	36.0	41.7	42.3

### 2.2 Pasta Making

The ingredients were weighed according to the formulation and mixed in a pasta machine Pasta Maker HR2375‐200 W (Koninklijke Philips N.V., Netherlands) for a total time of 6 min under standard automatic conditions. The pasta, directly extruded into sheets with a thickness of 2 mm, was rolled using a rolling machine PastaPresto NSP 85 W (Imperia & Monferrina S.P.A., Italy) until reaching a thickness of 1 mm. The sheets were pasteurized in an industrial line at 90°C ± 1°C for 2 min and then cut into *“tagliatelle”* with a width of 5 mm. Following heat treatment, the product was cooled to 5°C within 2 h. Pasta samples from different batches were packaged in plastic bags under modified atmospheric conditions. Each package, containing 100 g of product, was stored at 4°C until testing. For each test, true replicates consist of samples drawn from different batches.

### 2.3 Chemical Composition

Moisture was determined according to AACC Method 44–15.02 (AACC International 2009f). Proteins were quantified by the Kjeldahl method (AACC Method 46–11.02, conversion factor 6.25) (AACC International 2009c). Lipids were determined by Soxhlet extraction using petroleum ether (AACC Method 30–25.01) (AACC International 2009b). Ashes were assessed according to AACC Method 08–01.01 (AACC International 2009a). Total dietary fiber (TDF) content was measured as in AACC Method 32–05.01 (AACC International 2009g) with the dedicated K‐TDFR kit (Megazyme, Wicklow, Ireland). Total starch was determined as the sum of digestible starch and resistant starch from the AACC Method 32–40.01 (AACC International 2009d) by means of the K‐RSTAR kit (Megazyme, Wicklow, Ireland). The total energy value (kcal/100 g) was determined using the following conversion factors: protein 4 kcal/g, carbohydrate 4 kcal/g, fat 9 kcal/g, and fiber 2 kcal/g. All chemical tests were conducted in duplicate (*n* = 2), with at least two technical repetitions per batch, over ground uncooked pasta samples.

### 2.4 Water Holding Capacity (WHC) of Pasta Ingredients

The gluten‐free mix used for the control sample (as seen in Table 1) and the *A. domesticus* flour employed for the enriched samples were tested for their ability to hold water. The determination was performed by mixing 1 g of gluten‐free mix or *A. domesticus* flour with 10 g of distilled water in pre‐weighed 50 mL centrifuge tubes. The samples were thoroughly mixed by vigorous shaking (roughly 200 strokes min^−1^) for 20 min to favor proper hydration, and then centrifuged at 3000 × g for 25 min at 25°C. The supernatants were collected into pre‐weighed glass beakers and dried at 105°C overnight in an oven, and the pellets were weighed. The WHC was calculated as follows:

(1) $$\mathrm{WHC}\;\left(g\;\text{water}/g\right)=\frac{m_{p}-m_{s}-m_{\mathrm{sol}}}{m_{s}-m_{\mathrm{sol}}}$$

where *m*_*s*_, *m*_*p*_, and *m*_*sol*_ are the weights of the sample, the pellet, and the components solubilized in the water, respectively.

Each determination was performed in triplicate (*n* = 3), with at least three repetitions per batch.

### 2.5 Determination of Pasta Quality Parameters

#### 2.5.1 Cooking Loss and Water Absorption

The optimal cooking time (OCT) was determined by boiling the samples in distilled water and evaluating the pasta characteristics every 30 s. The determination of the cooking endpoint was conducted according to the AACC's 66–50.01 method, i.e., the time when the opaque region of the sample core disappeared. Moreover, given the difficulty to assess this phenomenon in gluten free samples, where the opaque core is not always clearly visible, three trained panelists were asked to sensory confirm the “*al dente*” texture in all cases (Lucisano et al. 2012).

To determine water absorption (WA), pasta samples were cooked for the OCT and drained for exactly 5 min. The samples were then immediately weighed, and the weight increase, relative to the dry weight of the uncooked sample, was calculated and expressed as WA.

Cooking loss (CL), expressed as g solids/100 g of dry pasta, was assessed as the weight of the solids obtained from the pasta cooking water after drying in a forced‐air oven at 110°C, according to the AACC official method 66–50 (AACC International 2009e). All determinations were carried out on three different cooking batches (*n* = 3) each with at least three technical repetitions, using a 1:10 pasta‐water ratio.

#### 2.5.2 Cutting Test

The cutting textural tests for the uncooked and cooked pasta were conducted using a TA.XTPlus Texture Analyzer (Stable Micro Systems, Godalming, UK) supported with a 50 kg load cell. Several pieces of pasta (length of about 3 cm and width of about 0.5 cm) were placed alongside on the lower plate, and a knife‐like probe (HDP/LKB) was used to cut the samples (test speed: 0.5 mm s^−1^). Firmness was measured as the maximum force required to cut the samples. Data are the mean of at least ten technical repetitions from three independent samples (*n* = 3).

#### 2.5.3 Color

Color measurements of uncooked and cooked samples were performed using a CR‐300 Chroma Meter (Konica Minolta Sensing Americas Inc., Osaka, Japan) with a CIE Standard Illuminant D65 and a 10° standard observer angle. Parameters L*, a*, and b* representing the lightness and the chromaticity coordinates for red‐green and yellow‐blue components, respectively, were measured. Data was obtained from six measurement points (technical repetitions) over the surface of three independent replicates (*n* = 3). To assess color changes compared to the control, and the color changes after cooking, the following equation (Equation 2) was applied:

(2) $$∆E=\sqrt{{\left(L_{i}^{*}-L_{R}^{*}\right)}^{2}+{\left(a_{i}^{*}-a_{R}^{*}\right)}^{2}+{\left(b_{i}^{*}-b_{R}^{*}\right)}^{2}}$$

where *ΔE* is the total color difference and the *i* and *R* subindices for each L* a* b* parameter correspond to the *i*‐th sample and the reference sample (C or uncooked samples, as appropriate), respectively.

### 2.6 Starch Digestibility

An in vitro procedure for starch digestibility was performed on minced cooked pasta samples using the AACC Method 32–40.01 (AACC International 2009d). Experiments were performed using the K‐RSTAR assay kit (Megazyme, Wicklow, Ireland). Approximately 0.5 g of each sample were incubated with 3 mL of pancreatic α‐amylase (10 mg/mL) and amyloglucosidase (3 U/mL) solution for 0, 20, 120, 180 min at 37°C with agitation. Enzymes activity was stopped by adding 4 mL of 96% ethanol. Samples were centrifuged at 5000 rpm for 10 min, and the supernatant was collected. The pellet was resuspended in 8 mL of 50% ethanol and centrifuged two times. Supernatants were collected together. The supernatants were incubated at 50°C for 20 min with 330 U/mL amyloglucosidase solution.

The amount of released glucose was measured using the GOPOD assay kit (Megazyme, Wicklow, Ireland), and the hydrolysis rate was expressed as the percentage of hydrolyzed starch in function of the time. The digestible starch (DS) was further classified as rapid digestible starch (RDS), i.e., the starch that was hydrolyzed in the first 20 min of incubation, and slowly digestible starch (SDS), i.e., the starch that was hydrolyzed between 20 and 120 min of incubation.

For the resistant starch (RS) content, samples were incubated for 16 h and processed in the conditions described. The pellets were treated with 2 M KOH for 20 min under stirring in an ice‐water bath, and then incubated with 3300 U/mL for 30 min at 50°C. Then, RS was calculated using the GOPOD assay kit.

The data represent the mean of three independent digestions conducted on respective independent batches (*n* = 3). TS content was calculated as the sum of the DS and RS. For all determinations, four technical repetitions were performed for each independent batch (*n* = 3), and the results are expressed as g/100 g dry weight.

### 2.7 Amino Acids Profiles Determination

The amino acids from the raw materials (GF mix and *A. domesticus* flour) and the cooked pasta samples were analyzed after hydrolysis. For the determination of all the amino acids but cysteine and tryptophan, samples were hydrolyzed with hydrochloride acid 6 M at 105°C for 24 h. Cysteine was determined as the sum of cysteine and cystine after the reaction with 3,30‐dithiodipropionic acid, producing a mixed disulfide, which then underwent acid hydrolysis. Tryptophan was determined by barium hydroxide hydrolysis of the samples at 105°C for 24 h. After hydrolysis, the samples were neutralized with 8 M NaOH or 6 N HCl, as appropriate. Acid hydrolysates were filtered with 0.45 μm syringe filters, and alkaline hydrolysates were filtered with 0.22 μm syringe filters. A pre‐column derivatization step with the AccQTag Ultra Derivatization Kit was conducted.

An Agilent 1260 Infinity HPLC (Agilent Technologies, USA) equipped with reversed‐phase columns (CORTECS C18, 2.7 μm, 2.1 × 150 mm kept at 45°C for amino acids from acid hydrolysis; Xselect HSS T3, 5 μm; 4.6 × 250 mm at room temperature for tryptophan), and a diode array detector (Agilent 1260 Series, DAD VL+) was used. An AccQTag Ultra was used for HPLC standards. The separation was performed by an isocratic elution system consisting of 25 mM sodium acetate/acetonitrile (91:9) delivered at 0.9 mL/min and detected with a DAD at 280 nm. The experiments were conducted using two true replicates (*n* = 2).

### 2.8 Protein Digestibility Corrected by Amino Acid Score (PDCAAS)

Digestibility of proteins in the cricket flour, the gluten‐free premix, and the pasta samples were determined using the K‐PDCAAS assay kit (Megazyme, Wicklow, Ireland). Briefly, 500 mg of sample were mixed with 19 mL HCl 0.06 N in a 50 mL centrifuge tube, incubated 30 min at 37°C in shaking conditions. Then, 1 mL pepsin solution was added, and the tube was vigorously mixed and incubated for 60 min at 37°C under shaking conditions. 1 mL of 1 M Tris buffer pH 7.4 was added to adjust the suspension's pH to 7.4. Then, 200 μL of trypsin/chymotrypsin solution was added, and the samples were incubated 4 h at 37°C in a shaking water bath. Then, samples were heated in a 100°C water bath for 10 min. After cooling for 20 min, 4 mL of the sample were mixed with 1 mL of TCA 40% and let it decant overnight at 4°C. 1.75 mL of the supernatant was transferred into a 2 mL centrifuge tube and centrifuged 10 min at 15000 × g. Supernatant was diluted with 50 mM acetate buffer pH 5.5 (1:10 and 1:20), and the primary amine content of these solutions were quantified by the ninhydrin method. For this, 100 μL of each solution, were mixed with 50 μL ninhydrin (2%) in a 96‐well plate, incubated 35 min at 70°C under shaking, and cooled for 10 min. 150 μL of ethanol (50%) was added, and the samples were read at 570 nm in an Agilent BioTek Synergy HTX multimode reader (Agilent Technologies Inc., USA). An L‐glycine standard curve, a blank, and a casein control were also measured under the same conditions. The experiments were conducted using three independent batches (true replicates, *n* = 3), each assayed in duplicate (two technical repetitions).

### 2.9 Visual Appearance and Smell Perception

The visual appearance and smell perception of uncooked and cooked pasta samples were determined by non‐trained panelists. Randomly codified samples were presented to the panelists (*n* = 38 for the uncooked samples; *n* = 26 for the cooked samples) in transparent containers. Panelists were instructed to indicate their perception of each sample by selecting one of three symbols representing a favorable, neutral, or negative evaluation (Hidalgo et al. 2023). The uncooked and cooked samples were evaluated in two independent sessions conducted on different days. The cooked samples were presented warm to the panelists in open glass containers. Several cooked batches were prepared and evaluated within a short time to avoid differences due to cooling and dehydration of the samples. The panelist population was drawn from the Agripolis campus of the Università degli Studi di Padova and consisted of students and professionals in food technology, veterinary science, forestry, and agronomy, ranging in age from 20 to 65 years. All participants were aware of the presence of insect‐based ingredients in the tested samples.

The forms used to acquire signed informed consents from the participants and the accompanying instruction documents, have been approved by the Committee for Research Ethics of the Department DAFNAE at the University of Padova, which deemed them compliant with the current legislative guidelines, as stated in the classified Ethics Committee Opinion Protocol number: 0005229 of Oct. 10th, 2025, UOR: D160000, Class. II/4.

### 2.10 Statistical Analysis

Pasta samples from independent batches were treated as true replicates. At least two batches were tested in each assay. From each batch, at least two technical repetitions were conducted. Data is presented as mean ± standard deviation (SD). Statistical analyses were performed using one‐way analysis of variance (ANOVA) followed by Fisher's LSD post hoc test in InfoStat v2018 (Di Rienzo et al. 2018), based on true replicates, at a significance level of 5% (*α* = 0.05).

## 3 Results and Discussion

### 3.1 Chemical Composition

The chemical composition of the uncooked gluten‐free pasta samples is presented in Table 2. The data shows a significant increase in the nitrogen and protein contents for both enriched samples. This increase was accompanied by a significant decrease in the lipid and carbohydrate content, and an increase in the mineral content (ashes). Interestingly, the amount of total dietary fiber was not modified, even when insect flours are known for the presence of undigestible chitin.

**TABLE 2:** Chemical composition of uncooked pasta samples.

	Control	AD5.8	AD10
Moisture	43.1 ± 0.0 b	46.0 ± 0.2 a	46.8 ± 0.3 a
Lipids	3.9 ± 0.1 a	2.6 ± 0.0 c	2.9 ± 0.0 b
Carbohydrates	46.9 ± 0.4 a	40.8 ± 0.2 b	37.5 ± 0.6 b
Ashes	0.26 ± 0.08 b	0.42 ± 0.09 ab	0.61 ± 0.04 a
Total dietary fiber	2.4 ± 0.8 a	2.9 ± 0.1 a	2.4 ± 0.4 a
Total nitrogen	0.62 ± 0.01 c	1.29 ± 0.01 b	1.73 ± 0.01 a
Crude protein†	3.9 ± 0.1 c	8.1 ± 0.1 b	10.8 ± 0.1 a
Protein‡	3.1	6.2	8.6
Chitin§	NA	3.3	3.5

*Note:* Values are expressed as mean ± standard deviation (g/100 g w.b.). Carbohydrates calculated by difference. Different letters in the same row indicate significant differences (*n* = 2; *p* < 0.05).

^†^ from total nitrogen conversion (N‐to‐protein conversion factor 6.25).

^‡^ from amino acids analysis of raw ingredients by HPLC.

^§^ from nitrogen fractions analysis (N‐to‐chitin conversion factor 14.1). NA: not applicable.

As it is commonly discussed among insect‐based foods, there is an important gap between the protein content calculated by conventional methods, such as that coming from the conversion of the total nitrogen (Nt) content into proteins using the 6.25 Kjeldahl factor (crude protein), and the actual content of protein when the nitrogen coming from chitin (nonprotein nitrogen, Nnp) is subtracted from the calculus (Jonas‐Levi and Martinez 2017). This same issue also affects the TDF determination, as this method requires quantifying the nitrogen in the fiber residue and subtracting it from the final value, which critically underestimates the actual fiber content of the sample.

To address these issues, the protein nitrogen (Np), calculated from the amino acids contents of the raw ingredients determined by HPLC (Table S1 and Table S2), was employed to calculate the corrected protein content. Then, the Nnp was calculated by subtracting the Np from the Nt, and converted to chitin by using a N‐to‐chitin conversion factor of 14.1, assuming a typical chitin degree of acetylation of 0.9 (Toribio et al. 2024). The results are shown in Table 2.

As expected, the values of protein calculated from the Np were lower than those determined by the conventional method (Nt determination and further conversion to crude protein) due to the increasing content of Nnp, especially for the enriched samples. On the other hand, the results suggest an estimated chitin content of ~3.4% for both of the insect‐enriched samples.

According to the data, the amounts of protein in the samples would contribute with 7%, 15%, and 20% of the total energy content when using the crude protein values. Thus, according to the EC Regulation No 1924/2006 (European Parliament and Council 2006), the enriched gluten‐free pasta samples could be commercialized as “source of protein” in the case of AD5.8, and as “high protein” for AD10. However, when using the actual protein values calculated by the amino acid analysis, the contribution of the proteins to the total energy content drops to 5%, 12%, and 16% for the control, AD5.8, and AD10, respectively, so in this case both enriched samples would be considered as “source of protein”.

It should be noted that these values are presented for comparative purposes only, and do not in themselves establish eligibility for nutrition claims, which depends on jurisdiction‐specific regulatory interpretation. However, this matter represents a critical issue for the commercialization of insect‐based meals, as current regulations do not specify which protein quantification method should be used to substantiate nutrition claims. The lack of harmonized criteria creates uncertainty for manufacturers and regulators, and may compromise the consistency and reliability of labeling practices (Boulos et al. 2020; Jonas‐Levi and Martinez 2017). However, European authorities emphasize the lack of scientific evidence to support regulatory changes, and platforms specialized in insects for food and feed, such as IPIFF, still recommend adherence to the general 6.25 conversion factor until further research is conducted (EFSA 2021). Clear guidance is therefore needed to ensure that protein declarations in insect‐based foods are both scientifically robust and aligned with consumer protection standards.

### 3.2 Cooking Quality

#### 3.2.1 Cooking Time, Water Absorption and Cooking Loss

**FIGURE 1:**
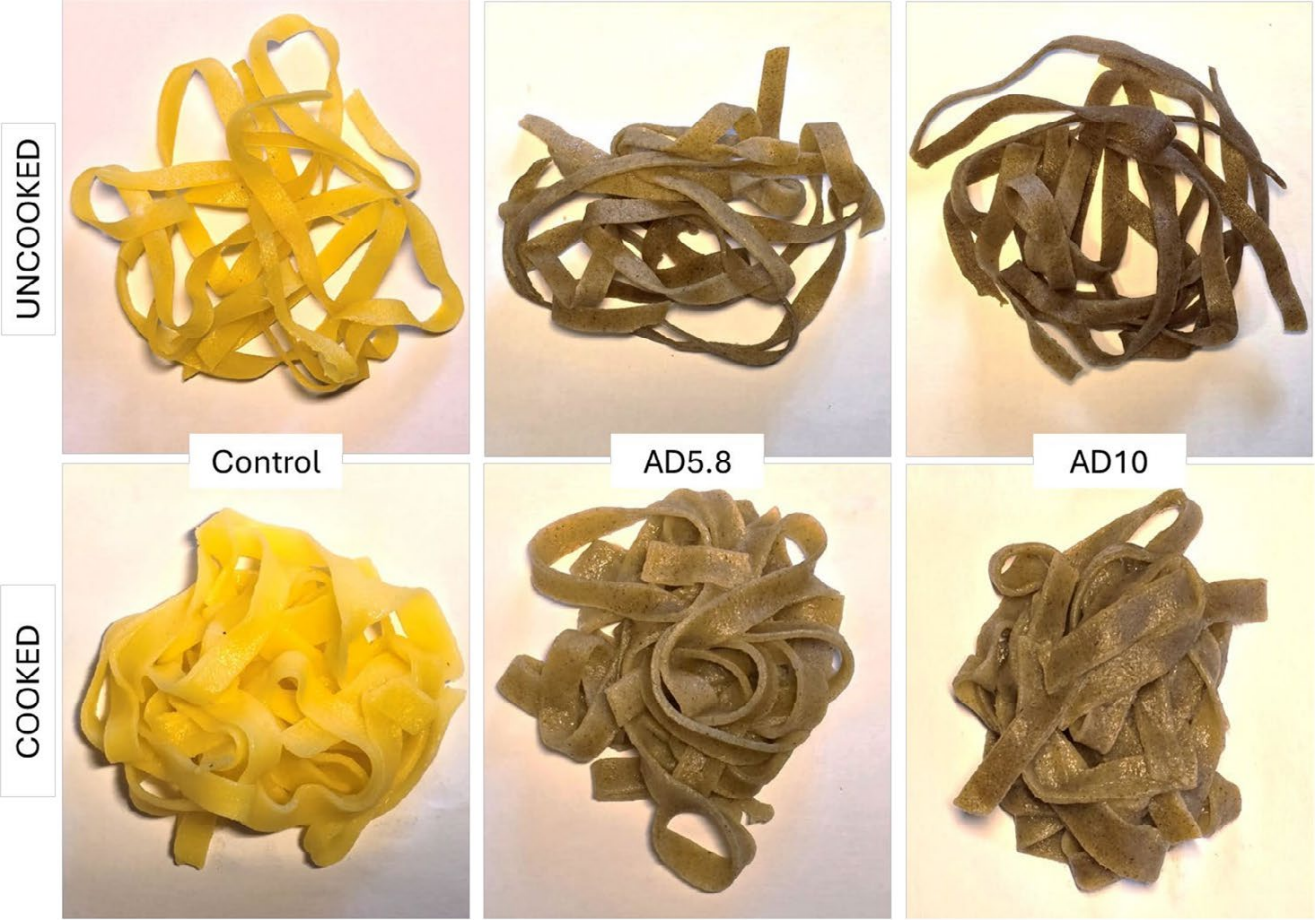
Images of gluten‐free fresh pasta samples before and after cooking.

The OCT was determined for all samples. The appearance of the uncooked and cooked samples is shown in Figure 1.

The control sample required a significantly longer cooking time (255 ± 28 s) compared to the enriched samples (150 ± 24 s), which showed no significant differences among themselves. For a given type of pasta, such as *“tagliatelle”*, the cooking time is mostly related to the hydration kinetics of their ingredients, which is greatly influenced by the structure of the matrix and the amount and gelatinization properties of the starch (Chaudhary et al. 2025). There are still very few published studies reporting on the influence of insect flour in the cooking time of gluten‐free pasta. Among these studies, no differences were found in the OCT of gluten‐free millet pasta enriched with 5% and 10% *G. bimaculatus* powder (Jakab et al. 2020). Despite the lack of studies addressing gluten‐free pasta, published research on the gluten‐containing counterparts showed that the OCT is generally increased with the addition of insect‐based ingredients (Çabuk and Yılmaz 2020; Duda et al. 2019), with some exceptions where no differences were found (Biró et al. 2019; Ho et al. 2022a).

**FIGURE 2:**
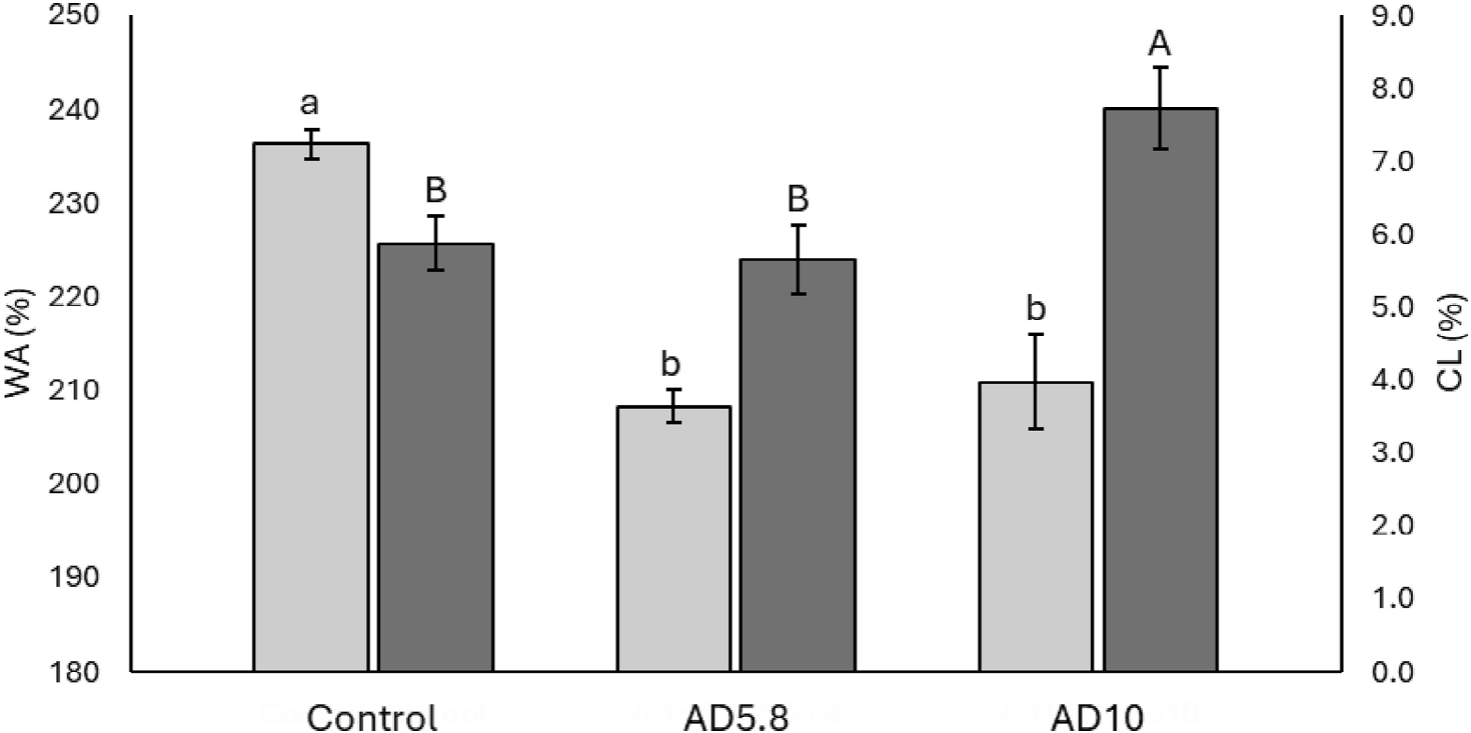
Water absorption and cooking loss of gluten‐free pasta. Different lowercase letters indicate significant differences for WA (*n* = 3; *p* < 0.05); different capital letters indicate significant differences for CL (*n* = 3; *p* < 0.05).

Similar outcomes were found for the WA (Figure 2). The control was characterized by a higher water absorption in comparison with the enriched samples (*p* < 0.05), which showed no differences between them (*p* > 0.05). This indicates that the enrichment strategy used to formulate the samples partially reduces the ability of the product to absorb water during cooking, even if the water holding capacity of the cricket flour is reported to be higher than 2 g water/g flour by different authors (Gantner et al. 2024; Han et al. 2023). This is attributed to the dilution of the starchy fraction in the formulations of the enriched samples, since the gluten‐free mix employed for the pasta production had a WHC of 2.81 ± 0.06 g water/g, while the *A. domesticus* flour had a significantly lower value of 1.94 ± 0.02 g water/g.

On the other hand, no significant differences were found between the CL of the control and the AD5.8 samples (*p* > 0.05). Conversely, AD10 showed a significant but slight increase in this parameter (*p* < 0.05) (Figure 2), indicating that increasing the proportion of protein at the expense of starch and oil can lead to structural modifications of the pasta matrix that affects its ability to entrap solids. Similar results were found by Piazza et al. (2023) for wheat‐based spaghetti enriched with 10% silkworm powder or 10% silkworm protein extracts.

Other authors have reported that interactions between gelatinized starch and network‐ or gel‐forming proteins, particularly gluten proteins, albumins, and soy proteins, help entrap solids within the matrix (Aravind et al. 2012; Vázquez‐Mata et al. 2024). However, cricket flours tend to aggregate rather than to form gels when the flour‐to‐water ratio is below 10% (Han et al. 2023). Consequently, the results of the present study may be attributed to the reduced amount of gelatinized starch and the increased presence of non‐gelling proteins in the enriched samples. The change in protein, starch, and oil proportions likely hindered the formation of the starch‐protein network, allowing greater lixiviation of solids, especially when fewer gel‐forming proteins and less gelatinized starch were available, as in the AD10 formulation. Nevertheless, all samples exhibited CL values that can be considered low, since none of them exceeded the 8% threshold generally regarded as the limiting value in the pasta industry (Desai et al. 2018).

#### 3.2.2 Texture

**FIGURE 3:**
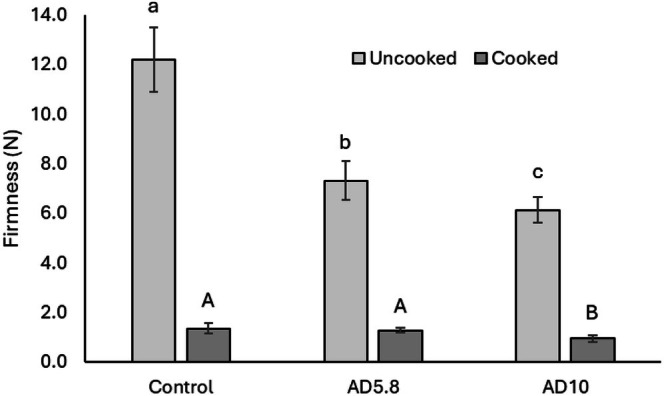
Firmness values of the uncooked and cooked pasta samples. Different lowercase letters indicate significant differences among uncooked formulations (*n* = 3; *p* < 0.05); Different capital letters indicate significant differences among cooked formulations (*n* = 3; *p* < 0.05).

The firmness of uncooked pasta samples progressively decreased with the increasing concentration of cricket flour (Figure 3). This is attributed to the dilution effect of the starchy components in the formulation, i.e., the change in the proportions of the different components in the characteristics of the starch‐protein matrix, as discussed above. In the enriched samples, the number of starch granules, which contributes to the firmness of the pasta matrix by acting as a filler, decreases, while the amount of proteins and lipids, which contributes to the softness of the product, increases. This is in agreement with previous research (Arp et al. 2018; Larrosa et al. 2015, 2016). On the other hand, the cooking process greatly decreased the firmness of the samples (Figure 3). However, after cooking, similar firmness values were found for the control and AD5.8. Although, AD10 presented a significant but slightly lower value of firmness in comparison with the other formulations. The results suggest that the cooking process was able to minimize the disparities in firmness values observed in the uncooked samples, and this could contribute to pair the acceptability of the enriched samples to that of the control one. Hormozi et al. (2025) reported a similar phenomenon for quinoa‐enriched noodles. In their study, the significant differences in the firmness values of the uncooked samples disappeared after cooking. Furthermore, the sensory tests results indicated that the control and the enriched samples (up to 20%) received the highest scores for parameters such as texture, mouthfeel, and overall acceptability.

#### 3.2.3 Color

**FIGURE 4:**
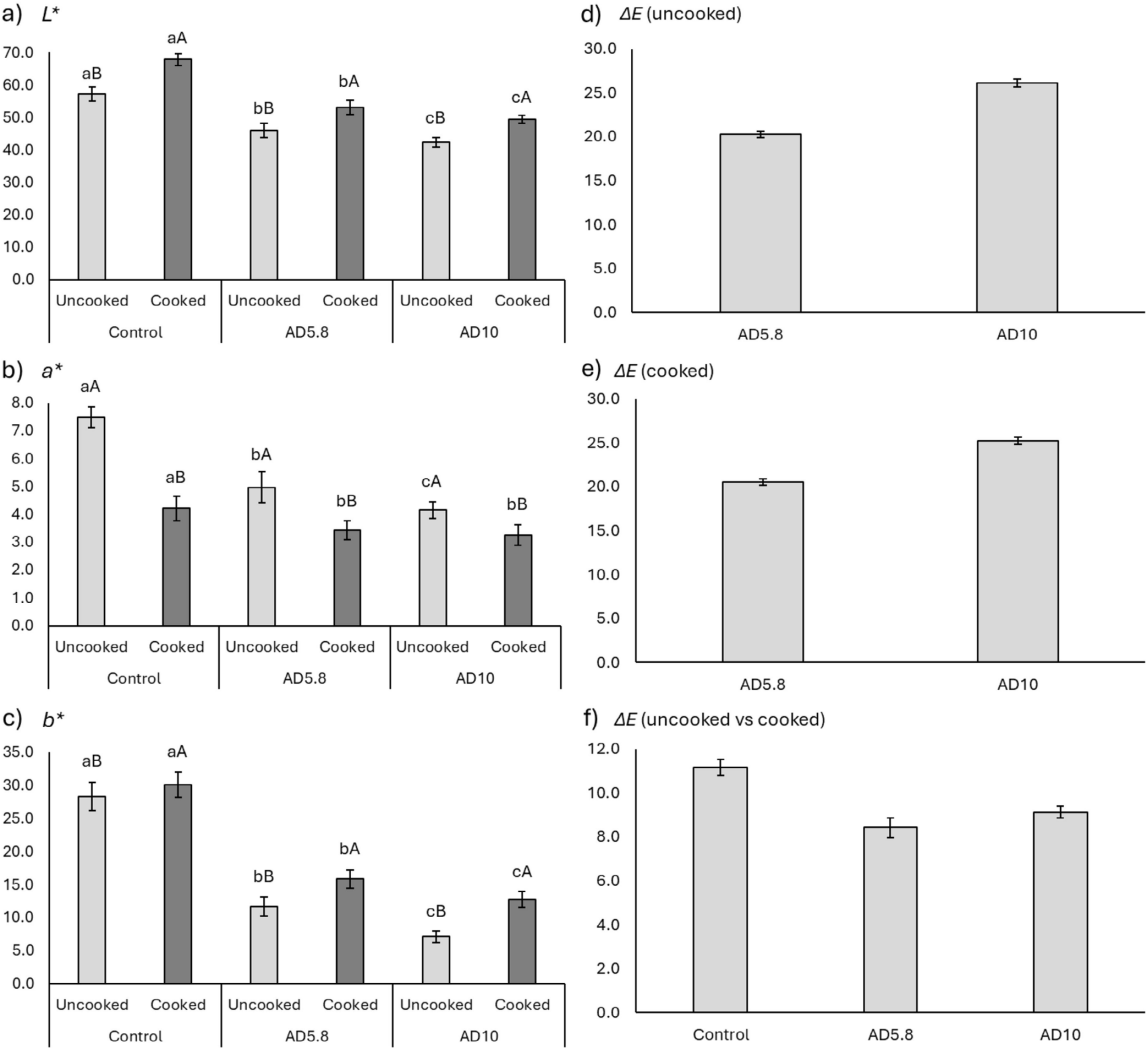
Color parameters of pasta samples (a) L*, (b) a*, (c) b*. For the L*a*b*parameters, different letters in the same series (uncooked or cooked), indicate significant differences among the different pasta formulations; different capital letters for the same pasta formulation indicate significant differences between uncooked and cooked samples (*n* = 3; *p* < 0.05). Color differences (d) *ΔE* of uncooked enriched samples respect to the control, (e) *ΔE* of cooked enriched samples respect to the control and (f) *ΔE* of samples before and after cooking.

The color was measured in the uncooked and cooked pasta samples. Results are shown in Figure 4. The values of L*, a*, and b* were significantly different for the uncooked samples among all the different formulations. With the addition of cricket flour, the uncooked samples became progressively darker, less red, and less yellow, developing a dark brownish hue (Figure 4a–c, light gray bars). Some authors have noted that such changes in insect‐enriched pasta may be perceived by consumers as whole wheat or integral pasta (Ho et al. 2022b).

After cooking, all samples showed significant changes. Samples became lighter, even less red, but more yellow (Figure 4a–c, dark gray bars). These changes could be associated with the cooking loss (Carpentieri et al. 2025), as wells as with the absorption of water, which would decrease the intensity of the color through the dilution of the colored components, leading to higher values of L*. Moreover, insects are usually a rich source of natural endogen and diet pigments such as melanin, sclerotin, carotenoids and riboflavin (Finke 2002; Inoue et al. 2025; Sukarman et al. 2023). Thus, the changes in the color parameters in the cooked pasta could be associated either with the dissolution of pigments into the cooking water (Carpentieri et al. 2025) and the degradation of such pigments, especially carotenoids, by the hydrothermal treatment (Ordóñez‐Santos and Martínez‐Girón 2020; Xiao et al. 2018).

The analysis of the *ΔE* parameter indicates that the color of both uncooked and cooked enriched samples was noticeably different from that of the control. The magnitude of these differences remained similar before and after cooking (Figure 4d,e), probably because the cooking process affected the color of the enriched samples to the same extent (Figure 4f). Similar results were reported by Carpentieri et al. (2025), who found no significant differences among the *ΔE* values of cooked mealworm‐enriched pasta in comparison with the uncooked ones.

### 3.3 Starch Digestibility

**FIGURE 5:**
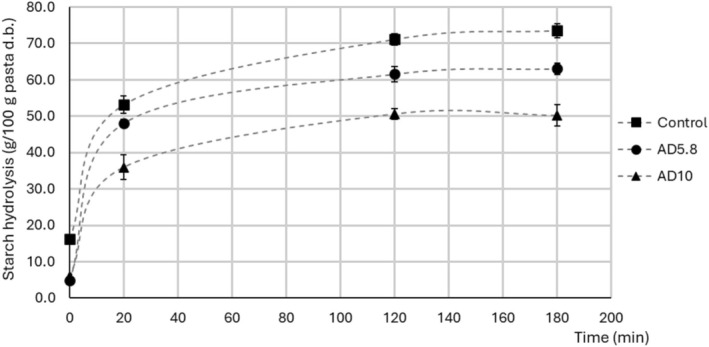
Starch digestibility of cooked pasta (*n* = 3; *p* < 0.05).

The results of the in vitro hydrolysis of starch for the cooked pasta samples are shown in Figure 5. Significant differences were found in the values of free glucose at the beginning of the hydrolysis (*t* = 0 min) among the control and the enriched samples. These values are attributed to free glucose in the gluten‐free premix used for the pasta preparation, that are diluted when the premix is replaced with cricket flour. The results show a significative decrease in the TS as the level of replacement increases, also due to the dilution of the starch by the replacement with the cricket flour.

The contents of RDS, SDS, and RS fractions were analyzed. The results showed that the control and AD10 samples had similar values for all the parameters, with RDS ranging from 59.9% to 61.1%, SDS from 29.2% to 29.7%, and RS from 10.9% to 9.2%, for the control and AD10, respectively. Conversely, AD5.8 exhibited higher values of RDS (71.1%), and lower values of SDS and RS (22.2% and 6.7%, respectively).

The differences in the hydrolysis profiles could be attributed to the combined effect of starch dilution and structural modifications of the pasta matrix when different levels of cricket flour are added. When no cricket flour is added (control), the pasta exhibits a dense and compact structure (high firmness) which is able to retain solids during cooking (low CL). The losses during cooking are mainly related to gelatinized starch leaching into the cooking water (Marti et al. 2010; Zhao et al. 2024). The dense structure of this sample would lead to lower diffusion of the amylolytic enzymes through the pasta matrix, slowing the digestion rate.

When adding low levels of cricket flour, such as in AD5.8, the matrix loses structural integrity (lower firmness). However, it is still able to retain its solids (lower CL). As a result, this sample has a softer matrix containing more gelatinized starch available for enzymatic attack, thus leading to enhanced enzyme diffusion and a higher rate of digestion. On the other hand, using higher levels of cricket flour (AD10) also led to a softer and less dense matrix. Nevertheless, in this case, significantly more solids were lost during cooking. Amylose leaching reduces the fraction of starch retained in the matrix, thereby lowering the amount available for rapid enzymatic hydrolysis inside the pasta structure.

### 3.4 Protein Digestibility

**FIGURE 6:**
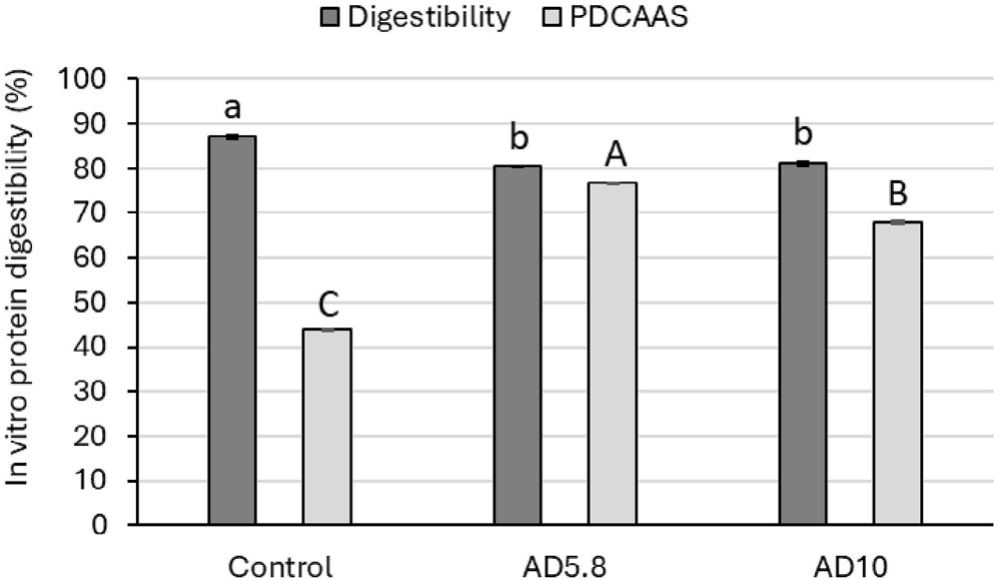
In vitro protein digestibility and PDCAAS of cooked pasta samples. Different lowercase letters indicate significant differences in in vitro protein digestibility (*n* = 3; *p* < 0.05); different capital letters indicate significant differences in PDCAAS (*n* = 3; *p* < 0.05).

The protein digestibility and PDCAAS values of the samples was determined, and the results are shown in Figure 6. All the cooked pasta samples exhibited an in vitro protein digestibility higher than 80%. However, the samples containing *A. domesticus* flour showed a slight but significant decrease in their digestibility values in comparison with the control, although both enriched samples were similar to each other. Different authors have reported similar values for a diversity of insect‐enriched samples such as rice noodles, triticale noodles, bread, snacks, and corn tortillas, ranging from 70% to 90% in vitro protein digestibility (Alvarez‐Barajas et al. 2023; Azzollini et al. 2018; Bottle et al. 2024; Li et al. 2025; Liu et al. 2025). Although different methods were used for the determinations, some authors have reported similar tendencies regarding the apparent slight decrease on the in vitro protein digestibility (Azzollini et al. 2018; Bottle et al. 2024).

The amino acids profiles of the cooked pasta samples indicate that the control is deficient in L‐lysine, with an amino acid score of 0.5044 (Table S3). Enriching the gluten‐free pasta with *A. domesticus* flour successfully increased the L‐lysine deficiency. Nevertheless, the enriched samples resulted slightly deficient in sulfur amino acids, exhibiting amino acids scores of 0.9549 (AD5.8) and 0.8366 (AD10) (Tables S4 and S5).

On the other hand, only few studies reported PDCAAS values for insect meals or insect‐enriched food samples. Azizi et al. (2025) reported PDCAAS values of 73 for cricket meals of the species *Gryllus assimilis* , while Jensen et al. (2019) found values ranging from 74 to 82 for *Tenebrio molitor* and different treated samples of *Alphitobius diaperinus* . Regarding insect‐enriched foods, Bottle et al. (2024) informed PDCAAS values of 45 for a control bread and values ranging from 64 to 69 for bread enriched with full‐fat, partially‐defatted, and defatted *A. domesticus* flours. The results indicate that using *A. domesticus* flour to enrich gluten‐free pasta formulations is an excellent strategy for increasing the protein quality of these kinds of products.

### 3.5 Visual Appearance and Smell

**FIGURE 7:**
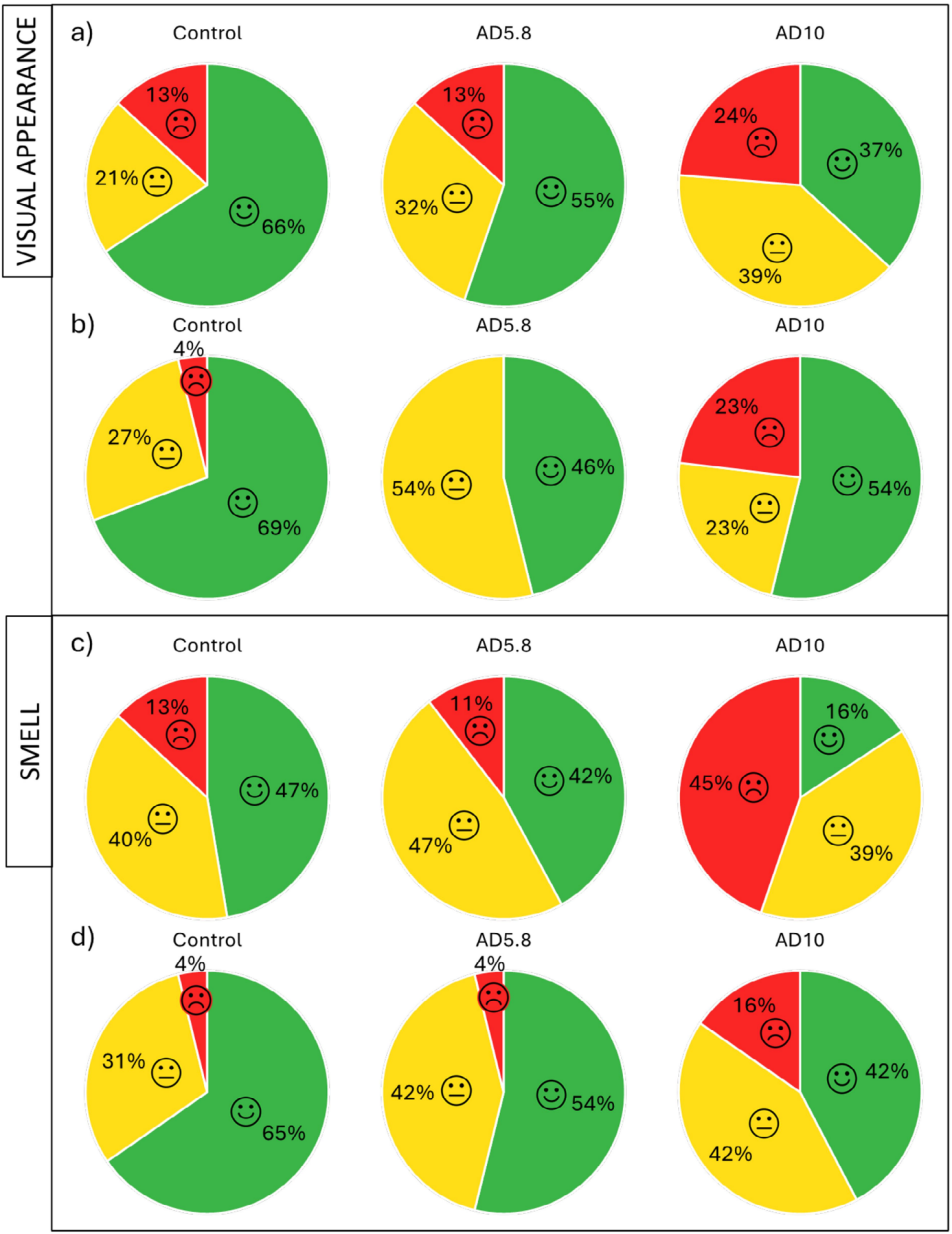
Visual appearance perception of (a) uncooked and (b) cooked pasta. Smell perception of (c) uncooked and (d) cooked pasta.

The visual appearance and smell of uncooked and cooked pasta samples were evaluated by consumers, and the results are shown in Figure 7. Overall, consumers gave positive responses for most of the samples, although some variability in the data can be noted. For the visual appearance of the uncooked samples, the percentage of favorable responses dropped from 66 to 37 as the level of *A. domesticus* flour increased (Figure 7a). However, it is worth noting that when the cricket flour was used at lower levels, the number of negative responses remained unchanged. This suggests that consumers tended to be doubtful about the appearance of the samples rather than disliking them. Nevertheless, increasing the level of cricket flour up to 10% had a negative influence on the visual perception of the uncooked samples.

On the other hand, when consumers evaluated the appearance of cooked samples (Figure 7b), the negative responses dropped markedly, giving way to more doubtful and positive answers for the control and AD5.8 samples. Although AD10 did not show changes in its negative responses, a shift from doubtful to positive answers was observed.

Regarding the smell, Figure 7c shows a trend similar to that of the visual appearance in the uncooked samples. However, in this case, the panelists were much more uncertain about how they felt toward the smell, even for the control sample. Similar favorable response profiles were observed for the control and AD5.8, whereas AD10 showed a markedly more negative outcome. These results indicate that the smell perception of uncooked pasta samples may be more critical than visual appearance when assessing these kinds of products. Comparable trends in the relative importance of visual and smell perceptions were found by Hidalgo et al. (2023) for semolina pasta enriched with protein extracts from *Bombyx mori* and *Hermetia illucens* .

Noticeably, when testing the smell of cooked samples, consumers responded more positively to all samples, including AD10, and the number of unfavorable responses decreased every case (Figure 7d). The results suggest that the liking profiles of the samples improve after cooking, particularly with respect to smell perception. Cooking reduces the volatile load of the samples, thereby softening the aroma intensity and producing more subtle profiles.

Even though AD10 exhibited up to 16% negative responses, it is worth noting that the assessment was conducted on plain samples. Pasta is typically consumed with sauces and other seasonings that provide more intense aroma profiles. Therefore, it is likely that more favorable responses toward this sample would be achieved under normal consumption conditions.

Given that taste was not assessed during the evaluation of the samples, the sensory analysis reported here is limited and should be considered exploratory. From this perspective, consumers were also asked about what the pasta aroma reminded them of. Most consumers tended to associate the aroma of cooked samples with concepts such as “whole‐grain”, “lentils”, “legumes”, “barley malt”, “protein pasta”, and “hazelnut”; whereas a smaller number associated the profiles with “feed”, “bran”, and “ammoniac”.

## 4 Conclusions

This study demonstrated that the partial substitution of corn starch and sunflower oil with *Acheta domesticus* flour in gluten‐free fresh pasta formulations can substantially improve the nutritional profile while maintaining acceptable technological performance. The enriched samples exhibited higher protein and mineral contents. Using calculus from amino acids count to correct values of protein by highlights the necessity of a consensus on protein determination of insect‐based foods to avoid overestimations and consequent misleading labeling. Cooking tests revealed shorter optimal cooking times, reduced water absorption, and cooking losses within industrially acceptable limits. Textural differences observed in uncooked pasta were minimized after cooking, supporting consumer acceptability, while color changes imparted a darker hue similar to those of whole‐grain products.

Starch digestibility profiles indicated that cricket flour can modify the hydrolysis kinetics depending on the level of substitution, likely due to structural changes of the pasta matrix. Enrichment improved lysine content and overall protein quality, as reflected in improved amino acid scores and PDCAAS values, although sulfur amino acids became limiting at higher inclusion levels.

While uncooked samples with higher levels of *A. domesticus* flour elicited more negative responses in visual and smell evaluations, whereas lower inclusion levels mainly produced neutral rather than negative responses. Cooking enhanced positive consumer responses to the pasta samples, particularly in terms of smell, by reducing volatile intensity and generating more subtle aroma profiles, highlighting its critical role in enhancing sensory acceptance of insect‐enriched pasta. However, taste was not evaluated, and thus the sensory analysis is limited and should be further explored.

Overall, the incorporation of cricket flour represents a promising strategy for functionalizing gluten‐free pasta, offering improved protein quality, reduced lipid content, and enhanced sustainability. Future research should address sensory evaluation, consumer perception, and regulatory frameworks to ensure accurate labeling and broader acceptance of insect‐enriched gluten‐free products.

## Author Contributions

**Gabriella Pasini:** conceptualization, funding acquisition, resources, project administration, supervision, writing – review and editing. **Alyssa Hidalgo:** writing – review and editing, project administration, funding acquisition. **Stefano Zardetto:** conceptualization, investigation, resources, data curation, writing – review and editing. **Carlos Gabriel Arp:** conceptualization, methodology, data curation, investigation, validation, formal analysis, visualization, writing – original draft, writing – review and editing.

## Funding

This research was funded by UE—NextGenerationEU cod. Progetto 2022JZ24J7—CUPC53D23005370006‐PNRR‐PRIN.

## Conflicts of Interest

The authors declare no conflicts of interest.

## Supporting information


**Table S1:** Amino acids profile of gluten‐free mix.
**Table S2:** Amino acids profile of A. domesticus flour (ADF).
**Table S3:** Amino acids profile of cooked Control pasta.
**Table S4:** Amino acids profile of cooked AD5.8 pasta.
**Table S5:** Amino acids profile of cooked AD10 pasta.

## Data Availability

The data that support the findings of this study are available from the corresponding author upon reasonable request.
