# Supplementary material for: Enrichment of Gluten‐Free Fresh Pasta With Cricket Flour: Technological and Nutritional Quality
Source: Food Sci Nutr. 2026 Aug 4;14(8):e72213. doi: 10.1002/fsn3.72213 (PMC13435262; doi:10.1002/fsn3.72213)
Supplement: Supplementary file 1 [file FSN3-14-e72213-s001.docx]

**Supplementary material**

**Enrichment of gluten-free pasta with cricket flour: technological and nutritional quality**

Table S1. Amino acids profile of gluten-free mix

| Amino Acid | GF Mix (g/100 g sample) | GF Mix (mg/g protein) | Ref. Protein* (mg/g protein) | Ratio |
| --- | --- | --- | --- | --- |
| L-Cys + L-Met | 0.1850 | 27.9854 | 25 | 1.1194 |
| L-Trp | 0.2750 | 41.5992 | 11 | 3.7817 |
| L-Asp/L-Asn | 0.5608 |  |  |  |
| L-Thr | 0.1610 | 24.3531 | 34 | 0.7163 |
| L-Ser | 0.3226 |  |  |  |
| L-Glu/L-Gln | 1.6640 |  |  |  |
| L-Pro | 0.4685 |  |  |  |
| L-Gly | 0.1965 |  |  |  |
| L-Ala | 0.4437 |  |  |  |
| L-Val | 0.2259 | 34.1819 | 35 | 0.9766 |
| L-Iso | 0.1533 | 23.1940 | 28 | 0.8284 |
| L-Leu | 0.7600 | 114.9766 | 66 | 1.7421 |
| L-Tyr + L-Phe | 0.4109 | 62.1591 | 63 | 0.9867 |
| L-Lys | 0.1633 | 24.7093 | 58 | 0.4260 |
| L-His | 0.1196 | 18.0870 | 19 | 0.9519 |
| L-Arg | 0.2224 |  |  |  |
| * FAO/WHO. (1991). Protein quality evaluation: Report of the Joint FAO/WHO Expert Consultation.  FAO Food and Nutrition Paper No. 51. Rome: FAO. | | | | |

Table S2. Amino acids profile of *A. domesticus* flour (ADF)

| Amino Acid | ADF (g/100 g sample) | ADF (mg/g protein) | Ref. Protein* (mg/g protein) | Ratio |
| --- | --- | --- | --- | --- |
| L-Cys + L-Met | 1.1946 | 17.3937 | 25 | 0.6957 |
| L-Trp | 3.4615 | 50.3997 | 11 | 4.5818 |
| L-Asp/L-Asn | 7.1340 |  |  |  |
| L-Thr | 2.7524 | 40.0764 | 34 | 1.1787 |
| L-Ser | 3.2913 |  |  |  |
| L-Glu/L-Gln | 10.3902 |  |  |  |
| L-Pro | 4.3548 |  |  |  |
| L-Gly | 3.7014 |  |  |  |
| L-Ala | 7.3766 |  |  |  |
| L-Val | 4.2272 | 61.5490 | 35 | 1.7585 |
| L-Iso | 2.9816 | 43.4130 | 28 | 1.5505 |
| L-Leu | 5.4885 | 79.9139 | 66 | 1.2108 |
| L-Tyr + L-Phe | 5.5709 | 81.1141 | 63 | 1.2875 |
| L-Lys | 5.3657 | 78.1261 | 58 | 1.3470 |
| L-His | 1.9663 | 28.6295 | 19 | 1.5068 |
| L-Arg | 4.4680 |  |  |  |
| * FAO/WHO. (1991). Protein quality evaluation: Report of the Joint FAO/WHO Expert Consultation.  FAO Food and Nutrition Paper No. 51. Rome: FAO. | | | | |

Table S3. Amino acids profile of cooked Control pasta.

| Amino Acid | Control (g/100 g sample) | Control (mg/g protein) | Ref. Protein* (mg/g protein) | Ratio |
| --- | --- | --- | --- | --- |
| L-Cys + L-Met | 0.2037 | 31.2857 | 25 | 1.2514 |
| L-Trp | 0.2748 | 42.2053 | 11 | 3.8368 |
| L-Asp/L-Asn | 0.5601 |  |  |  |
| L-Thr | 0.1866 | 28.6584 | 34 | 0.8429 |
| L-Ser | 0.3529 |  |  |  |
| L-Glu/L-Gln | 1.8394 |  |  |  |
| L-Pro | 0.5410 |  |  |  |
| L-Gly | 0.2196 |  |  |  |
| L-Ala | 0.5044 |  |  |  |
| L-Val | 0.2477 | 38.0517 | 35 | 1.0872 |
| L-Iso | 0.1734 | 26.6433 | 28 | 0.9515 |
| L-Leu | 0.8653 | 132.9200 | 66 | 2.0139 |
| L-Tyr + L-Phe | 0.4555 | 69.9710 | 63 | 1.1107 |
| L-Lys | 0.1905 | 29.2553 | 58 | 0.5044 |
| L-His | 0.1177 | 18.0748 | 19 | 0.9513 |
| L-Arg | 0.2459 |  |  |  |
| * FAO/WHO. (1991). Protein quality evaluation: Report of the Joint FAO/WHO Expert Consultation.  FAO Food and Nutrition Paper No. 51. Rome: FAO. | | | | |

Table S4. Amino acids profile of cooked AD5.8 pasta.

| Amino Acid | AD5.8 (g/100 g sample) | AD5.8 (mg/g protein) | Ref. Protein* (mg/g protein) | Ratio |
| --- | --- | --- | --- | --- |
| L-Cys + L-Met | 0.3349 | 23.8723 | 25 | 0.9549 |
| L-Trp | 0.6595 | 47.0036 | 11 | 4.2731 |
| L-Asp/L-Asn | 1.4263 |  |  |  |
| L-Thr | 0.5166 | 36.8234 | 34 | 1.0830 |
| L-Ser | 0.7483 |  |  |  |
| L-Glu/L-Gln | 2.9616 |  |  |  |
| L-Pro | 0.9947 |  |  |  |
| L-Gly | 0.6385 |  |  |  |
| L-Ala | 1.3142 |  |  |  |
| L-Val | 0.6759 | 48.1741 | 35 | 1.3764 |
| L-Iso | 0.4749 | 33.8462 | 28 | 1.2088 |
| L-Leu | 1.4389 | 102.5621 | 66 | 1.5540 |
| L-Tyr + L-Phe | 0.9499 | 67.7020 | 63 | 1.0746 |
| L-Lys | 0.8655 | 61.6882 | 58 | 1.0636 |
| L-His | 0.3347 | 23.8545 | 19 | 1.2555 |
| L-Arg | 0.7227 |  |  |  |
| * FAO/WHO. (1991). Protein quality evaluation: Report of the Joint FAO/WHO Expert Consultation.  FAO Food and Nutrition Paper No. 51. Rome: FAO. | | | | |

Table S5. Amino acids profile of cooked AD10 pasta.

| Amino Acid | AD10 (g/100 g sample) | AD10 (mg/g protein) | Ref. Protein* (mg/g protein) | Ratio |
| --- | --- | --- | --- | --- |
| L-Cys + L-Met | 0.4037 | 20.9157 | 25 | 0.8366 |
| L-Trp | 0.9359 | 48.4937 | 11 | 4.4085 |
| L-Asp/L-Asn | 2.0384 |  |  |  |
| L-Thr | 0.7224 | 37.4282 | 34 | 1.1008 |
| L-Ser | 0.9275 |  |  |  |
| L-Glu/L-Gln | 3.6521 |  |  |  |
| L-Pro | 1.2813 |  |  |  |
| L-Gly | 0.9213 |  |  |  |
| L-Ala | 1.8704 |  |  |  |
| L-Val | 1.1080 | 57.4111 | 35 | 1.6403 |
| L-Iso | 0.7930 | 41.0893 | 28 | 1.4675 |
| L-Leu | 1.8548 | 96.1054 | 66 | 1.4561 |
| L-Tyr + L-Phe | 1.3254 | 68.6758 | 63 | 1.0901 |
| L-Lys | 1.3230 | 68.5472 | 58 | 1.1818 |
| L-His | 0.4802 | 24.8801 | 19 | 1.3095 |
| L-Arg | 1.0727 |  |  |  |
| * FAO/WHO. (1991). Protein quality evaluation: Report of the Joint FAO/WHO Expert Consultation.  FAO Food and Nutrition Paper No. 51. Rome: FAO. | | | | |
